# Investigating the insecticidal potential of *Geomyces* (Myxotrichaceae: Helotiales) and *Mortierella* (Mortierellacea: Mortierellales) isolated from Antarctica

**DOI:** 10.1186/2193-1801-3-289

**Published:** 2014-06-09

**Authors:** Steven Edgington, Emma Thompson, Dave Moore, Kevin A Hughes, Paul Bridge

**Affiliations:** CABI UK-Centre, Bakeham Lane, Egham, Surrey, TW20 9TY UK; British Antarctic Survey, Natural Environment Research Council, High Cross, Madingley Road, Cambridge, CB3 0ET UK

**Keywords:** Cold, Fungus, Biological control, Host range, Insecticide

## Abstract

Fungi isolated from environmentally challenging habitats can have adaptations of potential value when developed as insect pest-controls. Fungal isolates collected from Antarctica, *Geomyces* sp. I, *Geomyces* sp. II, *Mortierella signyensis* and *M. alpina*, were investigated for (i) growth characteristics at 0–35°C, (ii) spore production at 10 and 20°C, (iii) viability following exposure to freezing temperatures, and (iv) insecticidal activity against waxmoths (*Galleria mellonella* L.), houseflies (*Musca domestica* L.), mealworms (*Tenebrio molitor* L.) and black vine weevils (*Otiorhynchus sulcatus* Fabricius). All isolates showed growth between 5–20°C, with some showing growth outside this range. *Geomyces* isolates sporulated over a wider range of conditions than the *Mortierella* isolates. Spore germination at 10°C was higher for *Geomyces* sp. II when this isolate was produced at 10 compared to 20°C (greatest difference 74.6 *vs* 32.7%). All isolates grew, with the exception of *M. alpina*, following exposure to −20°C for 4 weeks. Insecticidal investigations showed *M. alpina* and *M. signyensis* caused significant mortality of waxmoth and housefly larvae via injection and soil inoculation, and *M. alpina* caused significant mortality of housefly larvae via baiting; the *Geomyces* isolates had little lethal effect.

## Introduction

Sourcing insect-killing organisms from environmentally challenging habitats can uncover adaptations of value when developing these organisms as pest-control agents, e.g., cold-tolerant nematodes sourced from Scotland for black vine weevil control (Long et al. 2000) and this potential to find environmentally robust organisms has spurred an array of surveys from Sub-Saharan Africa (Shah et al. 1997) to within the polar circles (Vänninen 1996; Haukeland et al. 2006; Bridge and Worland 2008). In 2009 a project investigating cold-tolerant fungal isolates identified four strains collected from Antarctica (by British Antarctic Survey scientists) with enzyme profiles indicative of insecticidal potential (P. Bridge et al. unpublished results). The four isolates were characterised taxonomically: two were Zygomycetes in the genus *Mortierella* and two were Ascomycetes placed in *Geomyces*. Historically, most species of entomopathogenic fungi used as pest-controls are of the Ascomycete genera *Beauveria*, *Metarhizium*, *Lecanicillium* and *Isaria* (Bailey et al. 2010). The four candidates, all isolated from maritime Antarctica, showed high levels of chitinase and subtilisin proteases when grown in insect extract-based broths at low temperatures. Chitinases and subtilisins are key requirements for insecticidal activity as they provide the primary mechanisms for growth through both the exoskeleton and chitin of the insect (Clarkson et al. 1998). The psychrophilic *Geomyces* and *Mortierella* genera are widespread in Antarctic soils (Tosi et al. 2002; Hughes et al. 2003; Arenz et al. 2006) and it is likely that both genera have a role in decomposition and/or nutrient cycling; little, however, is known of their ability, if any, to kill invertebrates.

This paper investigates a number of characteristics relevant to the potential use of the four fungal isolates as mycoinsecticides in areas experiencing a temperate climate, including tolerance to colder temperatures and virulence against a number of common insect pests.

## Materials and methods

Cultures of *Geomyces* sp. I (IMI 403329; BAS CC373), *Geomyces* sp. II (IMI 403320; BAS CC364), *Mortierella signyensis* (IMI 398111) and *M. alpina* (IMI 403255; BAS CC299) were obtained from the British Antarctic Survey Culture Collection, held at CABI, UK. *Geomyces* sp. I and *Geomyces* sp. II were isolated originally from *Eucalyptus* bark and pine wood baits, respectively, suspended in sea water near Rothera Research Station, Adelaide Island, Antarctic Peninsula (67° 34′ S, 68° 06′ W). *Mortierella signyensis* and *M. alpina* were isolated from soil samples on Signy Island, South Orkney Islands (60° 43′ S, 45° 36′ W).

### Temperature profiles

Petri dishes (9 cm diameter) of 20% Potato Dextrose Agar (PDA) were inoculated at a central point with an upturned, non-sporulating plug (4 mm diameter) of each fungal isolate. The plugs were originally collected (using a cork-borer) from 20% PDA dishes that had been at 10°C, in the dark, for 6 d. The dishes were sealed with Parafilm and incubated at 0, 5, 10, 15, 20, 25, 30 and 35 ± 1°C. Radial growth was measured every 2 d along two perpendicular axes which intersected at the centre of the plug. There were five dishes per isolate, at each temperature. The experiment was run for 10 d.

### Spore production and germination

Inocula for this study came from fungal cultures grown on 20% PDA for 6 wk at 10 ± 1°C. Each isolate produced spores that could be harvested by scraping with a microspatula into 0.05% Tween-80 + distilled water, with the exception of *M. alpina* which did not produce aerial spores – hence for this isolate only mycelium was used.

Petri dishes (5 cm diameter) of 20% PDA were inoculated with 40 μL fungal inoculum of *Geomyces* sp. I, *Geomyces* sp. II and *M. signyensis*, at a spore concentration of approximately 3 × 10^5^ spores mL^-1^, and with four equally spaced upturned mycelial plugs (4 mm diameter) of *M. alpina*. Spore inoculum was spread evenly across each dish. Dishes were sealed with Parafilm and left in darkness at 10 and 20 ± 1°C. Every week, for 6 wk, five dishes of each isolate were harvested by scraping the surface gently with a microspatula into 0.05% Tween-80 + distilled water (efficiency of harvesting was not determined but it is likely that the majority of spores were collected at each harvest). A spore count was done for each dish using a haemocytometer. A 50 μL sample of each suspension was then inoculated onto a Petri dish (9 cm diameter) of 20% PDA to assess germination. The dish was sealed and kept in darkness at 10 ± 1°C for 24 h, after which time spore germination was assessed under a light microscope (×400 magnification). Spores were classified as germinated if the length of the germ tube was longer than the length of the spore. When possible, at least 200 spores were counted on each dish. There were five counts per treatment (representing one count from each harvested dish).

### Freeze tolerance

*Geomyces* spores and *Mortierella* plugs were used in this study. All inocula were collected from 2-wk old 20% PDA culture dishes at 10 ± 1°C. Spore suspensions (100 μL volume) or plugs were put into 0.5 mL microtubes and kept at −20 ± 1°C. Samples were removed after 4 and 8 wk then kept at room temperature for 30 min to defrost. Each spore suspension was spread across a 20% PDA Petri dish (9 cm diameter), which was then sealed and kept at 20 ± 1°C for 24 h, after which time spore germination was assessed under a light microscope (×400 magnification). Spores were classified as germinated if the length of the germ tube was longer than the length of the spore. When possible, at least 200 spores were counted on each dish. For the defrosted plugs, one plug was put upside down in the centre of a 20% PDA Petri dish (9 cm diameter), sealed and left at 20 ± 1°C for 1 wk, after which time radial growth was measured along two perpendicular axes, which intersected at the centre of the plug. Only plugs from 4 wk exposure were assessed. There were five samples (*i.e.*, five frozen microtubes) per fungal isolate, at each time assessment, with each originating from a separate original culture dish. Running concurrently to the experiment spore suspensions of *Geomyces* and plugs of *Mortierella* were put into microtubes and left at 5 ± 1°C (the laboratory standard for storage of these isolates) and assessed for germination or radial growth as above.

### Bioassays

#### Insect cultures

Waxmoths (*Galleria mellonella* L.), houseflies (*Musca domestica* L.), mealworms (*Tenebrio molitor* L.) and black vine weevils (*Otiorhynchus sulcatus* Fabricius) were used in this study. Waxmoths and mealworms were obtained from Live Foods Direct, Sheffield, UK; houseflies were obtained from Davies Angling, Staines, UK and black vine weevils were obtained from various potted plants in the glasshouses at CABI, UK.

#### Dipping

The fungal isolates were grown on 20% PDA culture plates (9 cm diameter) at 10 ± 1°C for 2 wk. Spores of *Geomyces* sp. I and sp. II were harvested using a microspatula and put into 0.5 mL aliquots of 0.05% Tween-80 + distilled water; concentrations were then adjusted to approximately 5 × 10^5^ spores/mL. *Mortierella signyensis* had not produced any spores after 2 wk so approximately 25% of mycelium from each plate was harvested and put into a 0.5 mL aliquot of 0.05% Tween-80 + distilled water. *Mortierella alpina* produced neither aerial spores nor aerial mycelium after 2 wk and was therefore excluded from this study. One late instar waxmoth larva was immersed in the fungal suspension for 5 s, placed onto filter paper within a bioassay chamber (2 cm^3^), then incubated at 10 ± 1°C, in darkness. Mortality was assessed every 1–2 d, for a maximum of 30 d. Cadavers were removed as soon as they were found. Control larvae were immersed in 0.5 mL 0.05% Tween-80 + distilled water. Ten larvae were treated per isolate (each in a fresh fungal suspension) and the trial was done four times.

#### Injection

The fungal isolates were grown on 20% PDA at 10 ± 1°C for a minimum of 2 wk. Spores were harvested from the dishes in 0.05% Tween-80 + distilled water (with the exception of *M. alpina* – which was subsequently excluded from this trial). Pathogenicity of the isolates was tested against larval stages of waxmoth, housefly and mealworm. Each insect was injected with 10 μL of fungal suspension at a concentration of approximately 1 × 10^6^ spores/mL, using a hypodermic syringe (outer gauge diameter approximately 1 mm). The insects were injected on the ventral surface, at approximately mid-body level. Control insects were injected with 10 μL of 0.05% Tween-80 + distilled water. Forty larvae were injected per isolate. The larvae were put in groups of 10 on filter paper within a Petri dish (9 cm diameter) and kept at 10 ± 1°C, in darkness. Larval mortality was assessed after 5 d. The trial was done three times, with the exception of the mealworm trial which was done twice.

#### Soil inoculation

*Mortierella signyensis* and *M. alpina* were grown on 20% PDA at 10 ± 1°C for approximately 12 wk. Petri dishes (9 cm diameter) were part-filled with double-sterilised (at 121°C for 15 min) John Innes compost (*ca* 20 g per dish). Four plugs (4 mm diameter) of each isolate were put into each dish. Control dishes received four plugs of 20% PDA. Ten mid to late instar waxmoth larvae or 20 late instar housefly larvae were put into each dish. The dishes were sealed and kept at 10 ± 1°C, in darkness. Insect mortality was assessed after 6 wk. There were five dishes per treatment, *i.e.* 50–100 larvae per treatment, depending on species. The trial was done three times.

#### Baiting

*Mortierella signyensis* and *M. alpina* were grown on 20% PDA at 10 ± 1°C for approximately 12 wk. Petri dishes (5 cm diameter) were part-filled with double-sterilised (at 121°C for 15 min) John Innes compost (*ca* 10 g per dish). Each dish received either two double-sterilised dry cat food biscuits (‘Hairball control – chicken’, Hill’s Pet Nutrition Inc, USA) or no biscuits. Three plugs (4 mm diameter) of each isolate were put into each dish. Control dishes received three plugs of 20% PDA. The dishes were sealed with Parafilm and incubated at 10 ± 1°C. After 7 d five late instar housefly larvae were put into each dish, which was then re-sealed and kept at 20 ± 1°C for 5 d. The houseflies were then removed and put into empty Petri dishes (5 cm diameter) at 20 ± 1°C, with mortality and adult emergence assessed after 2 wk. There were five baiting dishes per fungal isolate, *i.e.* 25 larvae per isolate. The trial was done three times.

### Data analysis

All statistical analyses were performed using Genstat 16th Edition (VSNI). Results from replicate trials were similar and were therefore combined for each fungal isolate. Percentage data (*i.e.* germination and mortality data) was arcsine transformed prior to analysis to improve homogeneity (the data presented here is pre-transformed data). One-way Analysis of Variance (ANOVA) with appropriate factors was used to investigate treatment effects, with Tukey’s test and *t*-test used to analyse relationships and differences between means. Differences between means were considered significant at *P* < 0.05.

## Results

### Temperature profiles

**Figure 1:**
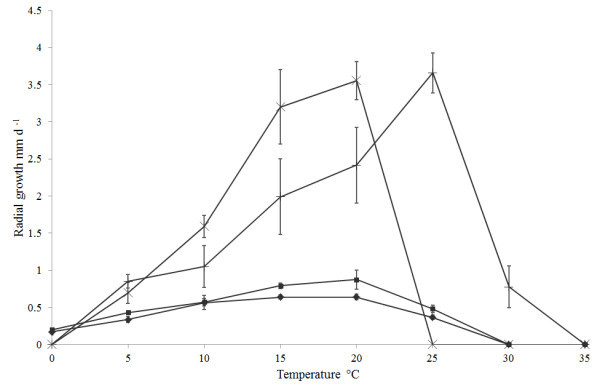
**The effect of temperature on the growth rate of** ***Geomyces*** **I (IMI 403329),** ***Geomyces*** **II (IMI 403320),** ***Mortierella signyensis*** **(IMI 398111) and** ***Mortierella alpina*** **(IMI 403255).** Vertical lines represent the 95% confidence intervals. ♦: *Geomyces* sp. I; ■: Geomyces sp. II; ×: *Mortierella signyensis*; +: *Mortierella alpina*.

Temperature profiles for the fungal isolates are shown in Figure 1. All isolates grew at 5, 10, 15, 20 and, with the exception *M. signyensis*, 25°C. *Mortierella alpina* was the only isolate with growth at 30°C with an extension rate of 0.8 mm d^-1^. There was measurable growth of both *Geomyces* isolates at 0°C, but no growth for either of the *Mortierella* isolates. Optimal growth temperatures were approximately 25°C for *M. alpina* and 20°C for the other isolates. There were significant differences between extension rates of the isolates at these optimum temperatures (*F* = 588.59, df = 3,16, *P* < 0.05); the *Mortierella* isolates had extension rates of 3.6 to 3.7 mm d^-1^ at their optima, which were significantly higher than those of *Geomyces* sp. I and *Geomyces* sp. II (0.64 to 0.88 mm d^-1^).

### Spore production and germination

**Figure 2:**
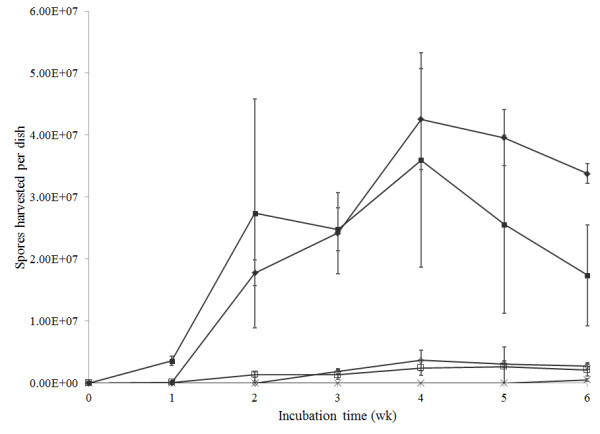
**Spore harvests of** ***Geomyces*** **I (IMI 403329),** ***Geomyces*** **II (IMI 403320),** ***Mortierella signyensis*** **(IMI 398111) and** ***Mortierella alpina*** **(IMI 403255) from culture dishes at 10 and 20 ± 1°C for 6 wk.** Vertical lines represent the 95% confidence intervals. ♦: 20°C *Geomyces* sp. I; ■: 20°C Geomyces sp. II; ×: 10°C *Mortierella signyensis*; ◊: 10°C *Geomyces* sp. I; □: 10°C *Geomyces* sp. II.

Spore yields from cultures over time and at different temperature can be seen in Figure 2. No spores of *M. alpina* were observed at any stage of the study and spores of *M. signyensis* were only found on 6-wk old cultures at 10°C. There were significant differences in yields between treatments (*F* = 51.06, df = 47,192, *P* < 0.05). *Geomyces* sp. II at 20°C was the first isolate to produce spores (after 1 wk), both *Geomyces* isolates produced spores after 2 wk at 10 and 20°C. Consistently more *Geomyces* spores were recovered from cultures at 20°C compared to 10°C. The highest yield was *Geomyces* sp. I after 4 wk at 20°C, which produced a mean of 4.2 × 10^7^ spores per dish (at 10°C this isolate yielded 3.7 × 10^6^ spores per dish at 4 wk). There were significantly fewer spores from *Geomyces* sp. II at 20°C at 6 wk *vs* 4 wk (1.7 *vs* 3.6 × 10^7^ spores per dish, respectively).

**Figure 3:**
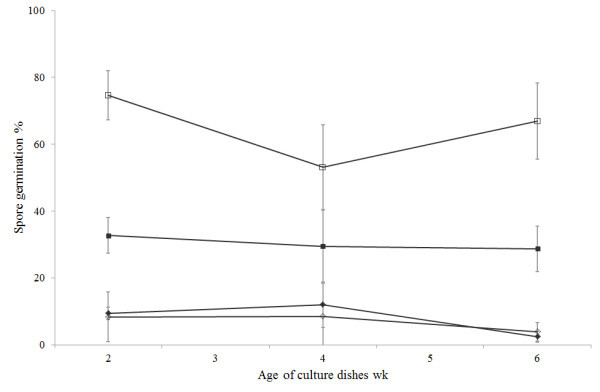
**Spore germination of** ***Geomyces*** **I (IMI 403329) and** ***Geomyces*** **II (IMI 403320) harvested from dishes at 10 and 20 ± 1°C and of different ages.** Vertical lines represent the 95% confidence intervals. ◊: 10°C *Geomyces* sp. I; □: 10°C *Geomyces* sp. II; ♦: 20°C *Geomyces* sp. I; ■: 20°C *Geomyces* sp. II.

Spore germination for the *Geomyces* isolates can be seen in Figure 3. There were significant differences in germination between treatments (*F* = 44.15, df = 11, 48, *P* < 0.05). Germination of *Geomyces* sp. II was significantly higher than *Geomyces* sp. I at each time and for each temperature (ranging from 29–75%) whilst *Geomyces* sp. I did not exceed 12% germination. Spores of *Geomyces* sp. II produced at 10°C had significantly higher germination at 10°C than those produced at 20°C, with the greatest difference being 75 *vs* 33% (for spores from 2-wk old dishes). Spores of *Geomyces* sp. I produced at 10 and 20°C had similar germination levels at 10°C.

### Freeze tolerance

Germination of *Geomyces* sp. I and *Geomyces* sp. II following exposure to −20 and 5°C can be seen in Table 1. There were significant differences in the germination of both isolates following exposure to −20°C for 4 wk (*Geomyces* sp. I: *F* = 214.2, df = 2,12, *P* < 0.05; *Geomyces* sp. II: *F* =142.1, df = 2,12, *P* < 0.05); falling from 89.5 to 17.0% and 97.4 to 40.7% after 4 wk at −20°C, for *Geomyces* sp. I and sp. II, respectively. After 8 wk at −20°C germination of *Geomyces* sp. I had fallen to 4.7%; there was no further reduction in the germination of *Geomyces* sp. II. Germination of both isolates was > 96% after 8 wk at 5°C. There were significant differences in radial growth of *M. signyensis* and *M. alpina* following exposure to −20 and 5°C (*F* = 58.6, df = 3,36, *P* < 0.05), both isolates had significantly lower levels of growth following 4 wk at −20°C *vs* 5°C (*M. signyensis*: *t* = 4.37, df = 18, *P* < 0.05; *M. alpina*: *t* = 3.48, df = 18, *P* < 0.05). Radial growth of *M. signyensis* totalled 12.2 ± 0.5 mm following −20°C exposure, compared to 20.7 ± 0.9 mm at 5°C; there was no growth of *M. alpina* after exposure to −20°C, compared to 15.2 ± 1.5 mm at 5°C.

**Table 1:** **Spore germination of** ***Geomyces*** **I (IMI 403329) and** ***Geomyces*** **II (IMI 403320) and vegetative radial growth of** ***Mortierella signyensis*** **(IMI 398111) and** ***Mortierella alpina*** **(IMI 403255) following storage at −20 and 5 ± 1°C, for 0 to 8 weeks (mean ± SE)**

Isolate	Temperature (°C)	Germination% (mean ± SE)			Radial growth mm (mean ± SE)
		0 wk	4 wk	8 wk	4 wk
*Geomyces* I	5	89.5 ± 1.2^a^	83.4 ± 1.8^a^	97.8 ± 0.6^b^	
	−20	89.5 ± 1.2^a^	17.0 ± 3.5^b^	4.7 ± 2.4^c^	
*Geomyces* II	5	97.4 ± 1.2^a^	90.7 ± 1.6^b^	99.4 ± 0.3^a^	
	−20	97.4 ± 1.2^a^	40.7 ± 3.7^b^	45.2 ± 4.2^b^	
*Mortierella signyensis*	5				20.7 ± 0.9^a^
	−20				12.2 ± 0.5^b^
*Mortierella alpina*	5				15.2 ± 1.5^a^
	−20				0 ± 0^b^

For each *Geomyces* isolate, figures with the same letter within each row are not significantly different; for each *Mortierella* isolate, figures with the same letter within each column are not significantly different (*P* > 0.05).

### Bioassays

#### Dipping

Mortality from the dipping study can be seen in Table 2. There were no significant differences in waxmoth mortality between treatments, including controls (*F* = 3.18, df = 3,15, *P* > 0.05), with mortality ranging from 13 (*M. signyensis*) to 35% (*Geomyces* I). Time until death was not significant between treatments (*F* = 0.38, df = 3,15, *P* > 0.05), ranging from a mean of (±S.E.) 17 (±4.5) to 23 (±1.6) d, from control to *Geomyces* 1, respectively.

**Table 2:** **Mortality of waxmoth (** ***Galleria mellonella*** **), housefly (** ***Musca domestica*** **), mealworm (** ***Tenebrio molitor*** **) and black vine weevil (** ***Otiorhynchus sulcatus*** **) larvae following inoculation with fungal isolates** ***Geomyces*** **I (IMI 403329),** ***Geomyces*** **II (IMI 403320),** ***Mortierella signyensis*** **(IMI 398111) and** ***Mortierella alpina*** **(IMI 403255)**

Inoculation		Isolate	Mortality%*			
			***Galleria mellonella***	***Musca domestica***	***Tenebrio molitor***	***Otiorhynchus sulcatus***
Dipping		*Geomyces* I	35.0 ± 8.7^a^	-	-	-
		*Geomyces* II	32.5 ± 4.8^a^	-	-	-
		*Mortierella signyensis*	12.6 ± 6.3^a^	-	-	-
		control	20.0 ± 4.1^a^	-	-	-
Injection		*Geomyces* I	8.3 ± 3.3^a^	29.2 ± 6.8^a^	10.0 ± 2.4^a^	-
		*Geomyces* II	1.7 ± 1.2^ab^	26.7 ± 6.5^a^	8.8 ± 2.0^a^	-
		*Mortierella signyensis*	94.2 ± 2.8^c^	62.5 ± 7.4^b^	67.5 ± 4.1^b^	-
		control	5.0 ± 3.2^ab^	15.0 ± 3.9^a^	7.5 ± 2.2^a^	-
Soil		*Mortierella signyensis*	0.4 ± 6.1^a^	38.3 ± 3.0^a^	-	6.0 ± 3.1^a^
		*Mortierella alpina*	50.7 ± 9.8^b^	33.0 ± 4.4^a^	-	2.0 ± 2.0^a^
		control	0.8 ± 0.6^a^	33.7 ± 0.7^a^	-	12.0 ± 6.1^a^
Baiting	food	*Mortierella signyensis*	-	34.7 ± 7.1^ab^	-	-
		*Mortierella alpina*	-	50.7 ± 7.1^b^	-	-
		control	-	25.3 ± 9.3^ab^	-	-
Baiting**	no food	*Mortierella signyensis*	-	6.7 ± 1.3^a^	-	-
		*Mortierella alpina*	-	9.3 ± 5.3^a^	-	-
		control	-	8.0 ± 4.6^a^	-	-

- = trial not done for this species; *mortality of *Musca domestica* was a function of non-emergence from puparia; **insects exposed to soil + fungus, with absence of food source (final fungal load may differ between dishes).

Inoculation was via dipping, injection, soil treatment and baiting. All trials were run at 10°C. Mortality was assessed after 5 d for injected insects and 4 wk for soil inoculation. For each insect and for each inoculation method, figures with the same letter within each column are not significantly different (*P* > 0.05).

#### Injection

Results from the injection bioassay can be seen in Table 2. There were significant differences in mortality between fungal treatments for waxmoths (*F* = 138.06, df = 4,55, *P* < 0.05), flies (*F* = 9.08, df = 4,55, *P* < 0.05) and mealworms (*F* = 29.66, df = 4,35, *P* < 0.05). *Mortierella signyensis* caused significant waxmoth, housefly and mealworm mortality, (*vs* control mortality) at 94.2 (*vs* 5), 62.5 (*vs* 15) and 67.5 (*vs* 7.5)% mortality for waxmoths, houseflies and mealworms, respectively; the other isolates caused no significant insect mortality.

#### Soil inoculation

Results from the soil inoculation bioassay can be seen in Table 2. There were significant differences in waxmoth mortality between treatments (*F* = 53.36, df = 2,42, *P* < 0.05). *Mortierella alpina* caused significant waxmoth mortality, at 50.7% *vs* <1% (control), *M. signyensis* had no effect on mortality. There were no significant differences in housefly mortality between treatments (*F* = 0.83, df = 2,42, *P* > 0.05), mortality ranged from 33 to 38.3%. There were no significant differences in black vine weevil mortality (*F* = 1.43, df = 2,27, *P* > 0.05), mortality ranged from 2 to 12%.

#### Baiting trial

Results from the baiting trial can be seen in Table 2. There were no significant differences in housefly mortality (as a function of non-emergence from puparia) between treatments for the food trial, and also the no food trial (food: *F* = 1.55, df = 2,42, *P* > 0.05; no food: *F* = 0.05, df = 2,42, *P* > 0.05). When results from the food and no food trials were analysed together (*F* = 5.84, df = 5,84, *P* < 0.05) there was a significant difference in housefly mortality between *M. alpina* food and *M. alpina* no food, at 50.7 *vs* 9.3%, respectively.

## Discussion

Surveys of Antarctic soils have revealed a number of fungal genera that have shown insecticidal activity in other environments, including the entomopathogenic hyphomycetes *Lecanicillium lecanii* and *Beauveria bassiana* (Mahaney et al. 2001; Hughes et al. 2003; see also http://www.antarctica.ac.uk/bas_research/data/access/fungi). Whilst there is no history of *Geomyces* and *Mortierella* killing insects, species of *Mortierella* have been investigated as potential biological control agents against bacterial and oomycete plant pathogens (Tagawa et al. 2010; Lambe and Wills 1983) and some Antarctic strains of *M. alpina* have been shown to exhibit anti-microbial activity (Melo et al. 2013).

In the present study, *M. alpina* produced neither aerial spores nor aerial mycelium; only vegetative growth was observed which was always under the surface of the culture media. Therefore, bioassay results from *M. alpina* are not directly comparable with the other strains that did produce spores. In contrast, the *Geomyces* isolates produced abundant spores; although of course quantity of spores is not the only factor of importance when developing a mycoinsecticide, as spore quality (persistence, viability, *etc.*,) is also critical for success (Magan 2001). All isolates grew at 5°C and both *Geomyces* grew at 0°C, a reflection of the cold climatic conditions where the isolates were collected e.g., soil temperatures on Signy Island are typically below 0°C for around nine months of the year (Chambers 1966) (although the *Geomyces* were isolated from the sea sampling). Furthermore, three of the isolates grew after four weeks exposure to −20°C. Cold tolerance may be of particular relevance to the use of fungi against insect pests in temperate climates as some larvae cause considerable damage to plants during winter months. None of the isolates grew at 30°C and above, although some strains showed tolerance to incubation at 35°C. For outdoor use in temperate climates, lower temperature limit is one of the principal environmental constraints to most, if not all, existing mycoinsecticide fungi (e.g., *Beauveria* and *Metarhizium*) (Inglis et al. 2001). Whilst many of these fungal groups are mesophilic, and grow between 10–40°C, they may only produce significant growth between 18–30°C (Rangel et al. 2005). If temperatures go outside this range and fungal growth is reduced as a result, then infection by the pathogens may not occur. Temperature constraints can, to some degree, be obviated by formulation (Langewald et al. 1999) and by modifying culture conditions to change spore physiology (Lane et al. 1991). In the present study, spores of *Geomyces* sp. II germinated consistently better at 10°C when they were grown at 10°C, compared to those grown at 20°C. A number of studies have shown that depleted resources and abiotic stresses during the production process, including exposure to thermal extremes, can lead to increased levels of endogenous compounds such as trehalose, glycerol and erythritol, which allow metabolic activity of the organisms to remain unaffected at times of sub-optimal environmental conditions (Crowe et al. 1984; Hallsworth and Magan 1994; Davis et al. 2000). In the present study, the improved germination of spores at cooler conditions when isolates were cultured at 10 *vs* 20°C, may be a consequence of placing the fungus under thermal stress.

The natural host range of the test isolates (if indeed they do kill in nature, as opposed to being opportunistic degraders of already dead invertebrates) is unknown as they were obtained directly from soil and marine samples. Both genera are known to be widespread in the Antarctic, particularly in soil; *Mortierella* species have been reported in association with Antarctic invertebrates (Bridge and Denton 2007) and the isolate of *M. signyensis* used in the present study was derived from such samples. Coprophilous Zygomycetes have been isolated from insect cadavers in the sub-Antarctic (Bridge et al. 2008) and a novel species of *Arthrobotrys* capable of trapping springtails has been described from the maritime-Antarctic (Onofri and Tosi 1992). It would seem likely that a number of fungal/invertebrate associations may occur normally in the Antarctic environment. It is possible that the *M. signyensis* isolate of the present study may have insect-killing properties as waxmoths, houseflies and mealworms all had higher levels of mortality, compared to the controls, when injected with this isolate. Furthermore, waxmoths in soil that contained *M. alpina* also died. There appeared to be no contact action of the isolates on houseflies, but the baiting studies suggested that at least one isolate (*M. alpina*) could kill, if ingested. However, caution is needed when interpreting these results regarding insecticidal potential as facilitating the introduction of the fungus into the host *viz* injecting it, negates an important component of insecticidal activity, namely breaching the insect’s integument. Hyphomycetous fungi can cause insect death by depleting insect nutrients, by physical obstruction and by toxicosis (or a combination of all three) but it is not known how *M. alpina* and *M. signyensis* caused insect death. The presence of chitinases and subtilisin proteases (discovered during a previous study) may indicate an ability to penetrate the insect cuticle, which could facilitate pathogenesis. Fatty acids have a long history as insecticidal agents (e.g. Tattersfield and Gimingham 1927) and some strains of *Mortierella* produce significant amounts of fatty acids. *Mortierella alpina* is used for the commercial production of archidonic acid, an omega-6 fatty acid with antimicrobial properties (Huang et al. 2010) and such compounds could provide a further potential insecticidal process.

This study has examined the physiology of four fungal isolates obtained from the Antarctic region and their ability to kill insects. *Mortierella signyensis* showed significant kill of insects from three orders and significant kill was also shown via two methods of delivery. The mechanisms of kill, however, are not known, but do not appear to correspond with those of more conventional entomopathogenic genera such as *Metarhzium*. It is unclear whether any of the isolates may be of value as a low-temperature mycoinsecticide, however, the environmental characteristics of their geographical origin may make Antarctica a potential source of fungi for biological control or other biotechnological applications.
